# Cyanophage Infection in the Bloom-Forming Cyanobacteria *Microcystis aeruginosa* in Surface Freshwater

**DOI:** 10.1264/jsme2.ME12037

**Published:** 2012-10-05

**Authors:** Yukari Yoshida-Takashima, Mitsuhiro Yoshida, Hiroyuki Ogata, Keizo Nagasaki, Shingo Hiroishi, Takashi Yoshida

**Affiliations:** 1 Subsurface Geobiology Advanced Research (SUGAR) Team, Extremobiosphere Research Program, Institute of Biogeosciences, Japan Agency for Marine-Earth Science and Technology (JAMSTEC), 2–15 Natsushima-cho, Yokosuka, Kanagawa 237–0061, Japan; 2 Structural and Genomic Information Laboratory, CNRS-UMR7256, Aix-Marseille University, Mediterranean Institute of Microbiology (FR3479), 163 Avenue de Luminy, Case 934, 13288 Marseille Cedex 9, France; 3 National Research Institute of Inland Sea, Fisheries Research Agency, 2–17–5 Maruishi, Hatsukaichi, Hiroshima 739–0452, Japan; 4 Department of Marine Bioscience, Fukui Prefectural University, 1–1 Gakuen-cho, Obama, Fukui 917–0003, Japan; 5 Graduate School of Agriculture, Kyoto University, Kitashirakawa-Oiwake, Sakyo-ku, Kyoto 606–8502, Japan

**Keywords:** cyanobacteria, cyanophage, non-bleaching gene (*nblA*), phycobilisome, *Microcystis*

## Abstract

Host-like genes are often found in viral genomes. To date, multiple host-like genes involved in photosynthesis and the pentose phosphate pathway have been found in phages of marine cyanobacteria *Synechococcus* and *Prochlorococcus*. These gene products are predicted to redirect host metabolism to deoxynucleotide biosynthesis for phage replication while maintaining photosynthesis. A cyanophage, Ma-LMM01, infecting the toxic cyanobacterium *Microcystis aeruginosa*, was isolated from a eutrophic freshwater lake and assigned as a member of a new lineage of the *Myoviridae* family. The genome encodes a host-like NblA. Cyanobacterial NblA is known to be involved in the degradation of the major light harvesting complex, the phycobilisomes. Ma-LMM01 *nblA* gene showed an early expression pattern and was highly transcribed during phage infection. We speculate that the co-option of *nblA* into *Microcystis* phages provides a significant fitness advantage to phages by preventing photoinhibition during infection and possibly represents an important part of the co-evolutionary interactions between cyanobacteria and their phages.

## Introduction

Viruses are the most abundant biological entities on earth and are considered to be one of the most significant factors regulating the abundance, diversity and composition of host populations (49, 50). Viruses also play a major role in nutrient cycling and genetic transfer (50). Of these, cyanophages (viruses infecting cyanobacteria) are reported to be predominant in aquatic environments by metagenomic analysis (1). Cyanophages infecting marine cyanobacteria *Synechococcus* and *Prochlorococcus* often carry multiple “host-like genes” (more recently called auxiliary metabolic genes) involved in photosynthesis and the pentose phosphate pathway (30, 31, 44, 45, 47). A large proportion of marine cyanophages contain *psbA* encoding D1 protein located in the core of photosystem II (PSII) (48), and the phage *psbA* is expressed during infection in which photosynthetic activity is maintained even after the decline of the host’s photosynthesis gene expression (25). A recent study showed that an increase of the host NADPH/NADP ratio during infection was accompanied by a decrease of Calvin cycle activity and increases of the pentose phosphate pathway and light reaction activity (55). Therefore, the gene products are considered to function as redirection of host metabolisms from carbon fixation to deoxynucleotide biosynthesis for phage replication while maintaining photosynthetic activity (55).

**Fig. 1:**
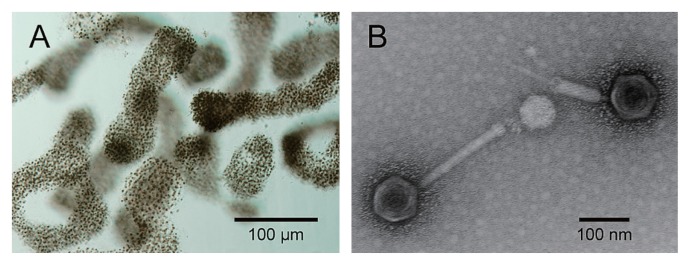
*Microcystis aeruginosa* and its cyanophage Ma-LMM01. (A) Optical micrograph of *Microcystis aeruginosa*; (B) transmission electron micrograph of negatively stained cyanophage Ma-LMM01.

We previously isolated a cyanophage Ma-LMM01 infecting a toxic strain of *Microcystis aeruginosa* from Lake Mikata, Fukui Prefecture, Japan (Fig. 1) (64). Since then, efforts have focused on understanding the interactions between *Microcystis* and their phages in freshwater environments. In this review, we summarize our current knowledge about the ecological role of cyanophages in *Microcystis* population dynamics, and the genome evolution of phages and *Microcystis*. In addition, we largely focus on a “host-like gene” of the Ma-LMM01 genome.

## Unique features of *Microcystis aeruginosa*

*Microcystis aeruginosa* frequently forms dense blooms in eutrophic fresh water throughout the world (3, 21). Some of the strains of *M. aeruginosa* produce hepatotoxins called “microcystins” (37, 38, 42, 56, 60, 63), which specifically inhibit eukaryotic protein phosphatase types 1 and 2A, and cause hepatocelluar carcinoma (27, 66). Due to acute poisoning, *M. aeruginosa* blooms sometimes cause the death of livestock and wildlife as well as humans (3, 18, 40).

Based on morphological criteria such as the cell arrangement in colonies and characteristics of mucilage, the genus *Microcystis* had been considered to be composed of multiple species: *M. aeruginosa*, *M. flos-aquae*, *M. ichthyoblabe*, *M. novacekii*, *M. viridis* and *M. wesenbergii* (22, 37, 38, 60, 63), so to clarify the taxonomic relationship among these morphospecies, genetic analyses were conducted. Most of these analyses demonstrated that the morphological features were not related to genetic relatedness (5, 56, 60, 63). Furthermore, DNA-DNA hybridization among *M. aeruginosa*, *M. novacekii* and *M. wesenbergii* demonstrated relatedness higher than 70%, confirming their synonymy based on the genetic criterion in bacterial taxonomy (22, 39). Although there are taxonomic aspects of unification of *Microcystis* species, recent field studies showed that *M. aeruginosa* is highly diverse at the intra-specific level, suggesting that multiple clonal types adaptable to various environmental conditions (“ecotypes”) may coexist within an *M. aeruginosa* population (23, 36, 53, 54, 58, 59).

The genomes of *M. aeruginosa* are remarkable for their abundance of repetitive sequences, including insertion sequences (ISs) (12, 19), which account for as high as 10.85% (gene content) of the *M. aeruginosa* NIES-843 genome (24), and these elements are predicted to bring plasticity into their genomes. Further, a recent report showed that the genome contains the largest number of defense genes (n=492), including the CRISPR (clustered regularly interspaced short palindromic repeats)-Cas (CRISPR-associated genes) systems, toxin-antitoxin system, and classical restriction- modification system among the 1,055 bacterial and archaeal genomes (28). Based on these observations, the occurrence of an evolutionary arms race between *M. aeruginosa* and its invading DNA, including plasmids and phages, is strongly suggested, which may have facilitated the diversity of this host-virus system.

## General features and ecology of Ma-LMM01

It has been suggested that there are morphologically and genetically diverse phages involved in *M. aeruginosa* (10, 17, 29, 57), but the *details* were not *known* except for Ma- LMM01 (51). The virion of Ma-LMM01 has an isometric head and a tail complex consisting of a central tube and a contractile sheath (Fig. 1B). Based on these morphological features, Ma-LMM01 most likely belongs to the group myovirus. Its latent period and burst size were estimated at 6–12 h and 50–120 infectious units cell^−1^, respectively.

We developed a real-time PCR assay system to quantify potential *M. aeruginosa*-infectious cyanophage (PMIC) abundance using primers targeting the sheath protein gene encoded by Ma-LMM01 (52, 61). Our field survey revealed that the abundance of PMIC was negatively correlated with that of *M. aeruginosa* (61). We also observed a temporal decline in the relative abundance of microcystin-producing genotypes within the *M. aeruginosa* population when PMIC numbers increased, suggesting that the cyanophage affected abundance as well as intra-species variation within the natural *M. aeruginosa* population; however, cyanophage infection most likely occurred in only a portion of the *M. aeruginosa* isolates tested. Thus, cyanophages may induce the replacement of phage-sensitive by phage-resistant populations, rather than having a quantitative impact on *M. aeruginosa* abundance (61, 62).

## Genome of Ma-LMM01

Ma-LMM01 has a linear, circularly permuted, and terminally redundant dsDNA genome 162,109 bp in length (65). The genome contains approximately 180 predicted protein coding genes and two tRNA genes. The majority of the predicted genes have no detectable homologues in the present databases and the genome showed no co-linearity with previously sequenced genomes of other *Myoviridae* including cyanomyoviruses; thus, Ma-LMM01 was just recently assigned as a member of a new lineage of the family *Myoviridae* (6, 23, 65). Ma-LMM01 possesses several genes, including transposase genes highly similar to the homologs found in cyanobacteria. This suggests the possible occurrence of relatively recent gene transfers between the Ma-LMM01- type cyanophage and its host(s) (23).

## Predicted function of Ma-LMM01 NblA

### Characterization of Ma-LMM01 NblA

The genome of Ma-LMM01 lacks homologs of photosynthetic genes that are highly prevalent in marine cyanophages. Instead, it has a “host-like” gene, *nblA*, which is essential for degradation of the major light-harvesting complexes, phycobilisomes (65).

Under high-intensity light conditions and nutrient depletion stress, the phycobilisomes are rapidly degraded, leading to a marked change in cellular color from the normal blue-green to yellow-green, referred to as bleaching or chlorosis (14). This is considered a mechanism to reduce the absorption of excess light energy under stress conditions and to provide amino acids for acclimation. The cyanobacterial *nblA* gene was first identified to be essential for this degradation process by analysis of a non-bleaching mutant (8, 43). The small polypeptide NblA (avg. Length 63 aa) is present in all phycobilisome-containing cyanobacteria, including *M. aeruginosa* (65), and Ma-LMM01 NblA shares 35% amino acid identity with the homologue in *M. aeruginosa*. There are two proposed explanations regarding the function of NblA in phycobilisome degradation. The first is that NblA may act as an adaptor protein that guides a ClpC-ClpP complex to phycobiliprotein disks in the rods of phycobilisomes, thereby initiating the degradation process (20). ClpC is an HSP100 chaperone partner of Clp protease (46). It has been previously shown that NblA binds via a highly conserved motif near its N terminus to ClpC and via another motif near its C terminus to phycobiliproteins (4, 20). The protein sequence determined from the Ma-LMM01 *nblA*-like gene had all of the highly conserved residues found in the cyanobacterial NblA protein sequences (65). Secondly, based on the crystal structures of NblA molecules from three cyanobacterial species, Dines *et al.* (11) proposed that NblA function is structure-dependent. NblA is made up of a helix-loop-helix motif, which is very similar to that of the phycobiliproteins, and loosens or disrupts the rod structure by structural mimicry, making them susceptible to proteolytic degradation. Ma-LMM01 NblA has a helix-loop-helix structure, and the structure is almost identical to cyanobacterial NblA (data not shown), suggesting a similar function of Ma- LMM01 NblA. To date, definitive conclusions as to whether NblA functions as the former and/or the latter proposed mechanisms have not been reached.

### Temporal pattern of transcription of Ma-LMM01 nblA

The prototypical myovirus, T4, has a temporally regulated transcription program that relies upon the sequential utilization of three classes of promoters: the early, middle, and late promoters (33). This feature is not always common in cyanophages; *e.g.* a marine cyanophage, S-PM2, which belongs to the T4 type myovirus, has only two temporal classes of transcription, early and late (7, 31). Previous analysis of the Ma-LMM01 genome indicated that the predicted ORFs are largely divided into two gene clusters (65): “UG region” and “LG region”. The UG region contains genes involved in DNA processing and nucleotide metabolism, while the LG region consists of genes involved in structural construction. The *nblA* gene belongs to UG region.

**Fig. 2:**
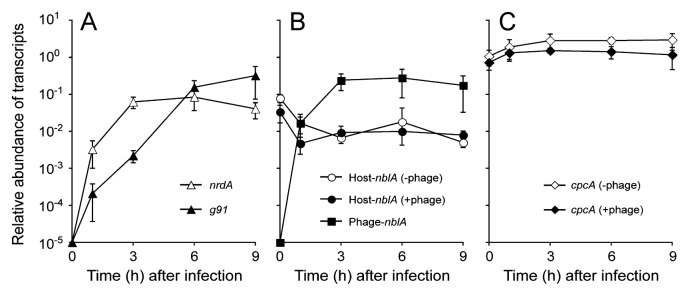
Expression profiles of phage and host genes. Transcript levels of phage- and host-encoded genes were determined using real-time RT-PCR during infection of *Microcystis aeruginosa* NIES-298 to Ma-LMM01. The *rnpB* gene encoding RNase P RNA of *M. aeruginosa* was used as an internal standard to normalize the transcription levels. Mean and standard deviation of three replicates are shown. (A) *nrdA* and *g91*, (B) host-*nblA* and phage-*nblA*, and (C) *cpcA*. Only the expression profiles from 0 to 9 h pi are shown since the host cells were lysed at 12 h pi.

When we monitored temporal changes in the level of these Ma-LMM01 transcripts during infection by quantitative real-time PCR (Supplemental Materials and supplemental Table 1), no phage transcript was detected immediately after infection (Fig. 2A). Then, transcripts of a UG region, *nrdA* (coding for ribonucleotide reductase alpha subunit) appeared 1 h post infection (pi), reached close to its maximum at 3 h pi and remained at the same level until lysis (Fig. 2A). On the other hand, the abundance of transcripts of a LG region gene, *g91* (coding for the contractile tail sheath protein), progressively increased, reaching its maximum at 9 h pi (Fig. 2A). The transcriptional pattern of *g91* was clearly distinguished from that of *nrdA*. This result suggests that Ma-LMM01 has at least two different temporal classes of transcripts; *i.e.*, early (*e.g. nrdA*) and late (*e.g. g91*) genes. However, the Ma-LMM01 genome has no homologues of T4 genes for reprogramming host RNA polymerase or phage σ factor required for late transcription (65). This suggests that Ma-LMM01 may use a particular mechanism mostly dependent on the host’s transcriptional machinery to regulate its transcription throughout infection. For instance, the binding efficiency of host RNA polymerase may differ for promoters between classes of phage genes. Alternatively, it is most probable that Ma-LMM01 possesses genes for yet unidentified transcription factors in addition to the housekeeping σ factor.

When we examined the transcription of both the host- and phage-encoded *nblA* genes, the transcripts of phage *nblA* appeared at 1 h pi, reached its maximum at 3 h pi, and remained constant until the end of infection (Fig. 2B). Phage *nblA* thus showed an early expression pattern akin to that of *nrdA*, raising the possibility that the encoded protein may start functioning at an early stage of infection. The transcriptional patterns of the host’s *nblA* were similar in both infected and uninfected cells (Fig. 2B), while phage *nblA* transcription was twenty-fold higher than the host’s *nblA* from 3 h pi. Although we have no direct evidence whether phage NblA is responsible for the degradation of host phycobiliproteins, this suggests that the phage-encoded NblA acts on this process more effectively and specifically than the host’s NblA. In the infected host cells, transcripts of the host’s *cpcA* encoding the alpha subunit of phycocyanin, which is the major component of the light harvesting antenna, stayed almost at the same level as that in uninfected host cells (Fig. 2C), suggesting that phage infection has no effect on phycocyanin synthesis in infected cells.

### Potential strategy of phage NblA

Even though light is the primary source of energy for photosynthesis, excessive light energy can damage photosynthetic machinery (*i.e.*, photoinhibition) (41). Photosynthetic organisms have thus evolved multiple mechanisms to avoid the photoinhibition process (41).

**Fig. 3:**
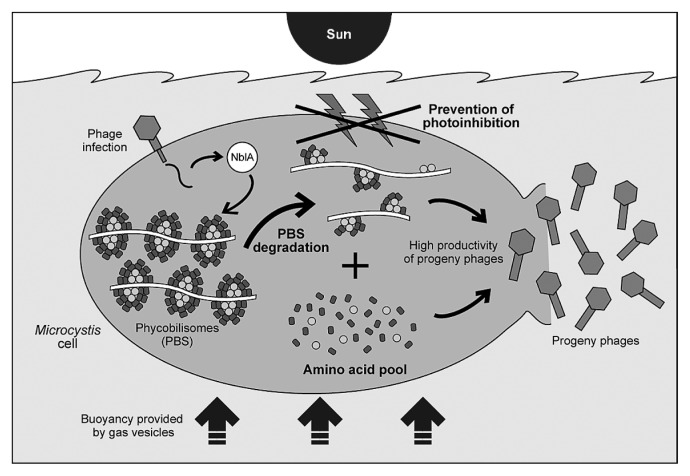
Schematic summarizing the potential strategy of cyanophage possessing *nblA* during infection of *Microcystis aeruginosa*. *M. aeruginosa* is commonly found in surface waters due to the buoyancy provided by the gas vesicles, and is intensively and frequently exposed to extensive light. Therefore, phage NblA might function to prevent photoinhibition caused by the absorption of excess light energy upon phage infection. In addition, the phage might use degraded phycobilisomes as an important source of amino acids for phage structural protein synthesis.

One of the mechanisms for preventing photoinhibition is found in the D1 repair cycle. D1 protein (PsbA) is a subunit of a heterodimer in the reaction center of PSII (32), and damaged D1 is rapidly removed and replaced by newly synthesized D1, thereby preventing photoinhibition (2). Recent studies further revealed that a large proportion of marine cyanophages contain *psbA* (48), and the phage *psbA* is expressed during infection where photosynthetic activity is maintained even after the decline of the host’s photosynthetic gene expression (7, 25, 26). Therefore, for cyanophages, it appears important to prevent photoinhibition during their infection and replication for rationally invading oxygenic phototrophs and effectively propagating themselves. In natural environments, *M. aeruginosa* floats near the surface (Fig. 3) (34); therefore, they are intensively and frequently exposed to extensive light, which may rapidly lead to photoinhibition upon phage infection (65). Recently, another example of *nblA* was found in phage PaV-LD infecting freshwater cyanobacterium *Planktothrix agardhii*, which also forms massive blooms on the water surface (13). Furthermore, phycobilisome degradation has been confirmed in a PaV-LD *nblA*-transfected strain of *Synechocystis* PCC6803 (13). These data support the hypothesis that phages infecting cyanobacteria inhabiting surface water habitats might be conferred with a fitness benefit by the degradation of phycobilisomes rather than the PSII repair cycle. Ma-LMM01 also has a long latent period where it may be crucial to decrease the absorption of excess light energy to prevent photoinhibition.

On the other hand, the benefit can also be interpreted as follows: the phage might use phycobilisomes as an important source of amino acids for phage structural protein synthesis (Fig. 3), since phycobilisomes constitute up to 50% of soluble protein in the cell (14). The availability of intracellular amino acids might provide a significant benefit for the replication of cyanophages during blooms, since extremely dense blooms of *Microcystis* cells can easily lead to local depletion of nutrient, *i.e.*, nitrogen starvation (9, 35, 56).

It is still unclear if *Microcystis* phages acquired the *nblA* gene by horizontal gene transfer from the cyanobacterial lineage, which led to the current hosts. Curiously, the *nblA* gene in the phage genome partially overlaps *nrdA* (ribonucleotide reductase alpha subunit), which is considered to be derived from *Microcystis* on the basis of their high level of sequence similarity. Since these two genes are widely separated in the host genome, it is presumed that *Microcystis* phage acquired these two genes from their hosts independently.

### Concluding remarks

Phages evolve by recombinational rearrangement of genes and the acquisition of metabolic and biosynthetic genes from their hosts (15, 16). Therefore, the presence of *nblA* in the *Microcystis* phage strongly suggests that co-opting this gene provided a significant fitness advantage to its survival tactics. Also, the co-options of host-like genes by cyanophages from not only marine but also freshwater implies diverse adaptive strategies for cyanophages to take over and control their host’s metabolism.

## Supplementary Material
