# Supplementary material for: Cyanophage Infection in the Bloom-Forming Cyanobacteria Microcystis aeruginosa in Surface Freshwater
Source: Microbes Environ. 2012 Oct 5;27(4):350–5. doi: 10.1264/jsme2.ME12037 (PMC4103541; doi:10.1264/jsme2.ME12037)
Supplement: Supplementary file 1 [file 27_350_s1.pdf]

Supplemental Materials

Materials and methods

The host strain, *M. aeruginosa* NIES-298 was obtained from the National Institute for Environmental Studies (NIES), Environmental Agency, Japan, maintained and grown using CB medium (Kasai, F., et al. 2004) under condition as previously described (Yoshida, T., et al. 2006). Exponentially growing cells were infected with Ma-LMM01 at a multiplicity of infection (MOI) of 0.01 as described previously (Yoshida, M., et al. 2010). The infection experiments were carried out in triplicate. The transcriptional analysis using real-time reverse transcription-PCR (RT-PCR) was conducted as described previously (Yoshida, M., et al. 2010). The primers used for the real-time RT-PCR are listed in Supplemental Table 1 with their annealing and extension temperatures. The *rnpB* gene encoding the RNase P RNA of *M. aeruginosa* was used as an internal standard to normalize the transcription levels. This housekeeping gene has been shown to serve as a reliable control even in phage-infected cells (Lindell, D., 2007; Yoshida, M., et al. 2010).

Kasai, F., M. Kawachi, M. Erata, and M. M. Watanabe. 2004. NIES-collection list of strains, 7th ed. The National Institution for Environmental Studies,

Tsukuba, Japan.

Lindell, D., et al. 2007. Genome-wide expression dynamics of a marine virus and host reveal features of co-evolution. Nature 449:83-86.

Takashima, Y., T. Yoshida, M. Yoshida, Y. Shirai, Y. Tomaru, Y. Takao, S. Hiroishi, and K. Nagasaki. 2007. Development and application of quantitative detection of cyanophages phylogenetically related to cyanophage Ma-LMM01 infecting *Microcystis aeruginosa* in fresh water. Microbes Environ. 22:207-213.

Yoshida, M., T. Yoshida, Y. Yoshida-Takashima, A. Kashima, and S. Hiroishi. 2010. Real-time PCR detection of host-mediated cyanophage gene transcripts during infection of a natural *Microcystis aeruginosa* population. Microbes Environ. 25:211-215.

Yoshida, T., Y. Takashima, Y. Tomaru, Y. Shirai, Y. Takao, S. Hiroishi, and K. Nagasaki. 2006. Isolation and characterization of a cyanophage infecting the toxic cyanobacterium *Microcystis aeruginosa*. Appl. Environ. Microbiol. 72:1239-1247.

Supplemental Table 1. Primers used in this study.

			Temperature (°C)		
Target gene	Primer name	Sequence (5'-3')	Annealing	Extension	References
nrdA	nrdARTF	GTCGCCGCTTCAATGCTG	58	84	this study
	nrdARTR	GCCTTAACCTTACGCCG			
nblA (phage)	nblARTF	GTGAGTGCCATTCCTGC	58	82	this study
	nblARTR	TCTTCTTGATGATAGCCGC			
nblA (host)	Mic-nblARTF	AACCGATCGAACTTTCCTTAG	58	78	this study
	Mic-nblARTR	GCGCTTGCTCCTGACTC			
cpcA	cpcARTF	GCCGCTAAAGTGTTGACCG	58	84	this study
	cpcARTR	GTCCGCCGCAAAGTTAG			
g91	sheathRTF	ACATCAGCGTTCGTTTCGG	58	84	Takashima Y. et al. 2007
	sheathRTR	CAATCTGGTTAGGTAGGTCG			
rnpB	rnpBRTF	GTGGGGAGCAAGGTGG	56	80	Yoshida M., et al. 2010
	rnpBRTR	CTTTTACCTTTGTTGGAATA			
